# PKM2 mediates the glycolytic response to matrix stiffness in lung fibroblasts

**DOI:** 10.3389/fcell.2026.1771078

**Published:** 2026-05-01

**Authors:** Yuan Wang, Ruihan Hu, Xu Zhang, George Cai, Qiang Ding, Pulin Che

**Affiliations:** 1 Department of Anesthesiology and Perioperative Medicine, Division of Molecular and Translational Biomedicine, University of Alabama at Birmingham, Birmingham, AL, United States; 2 Department of Cardiovascular Medicine, Guiqian International Hospital, Guiyang, China

**Keywords:** fibroblast activation, glycolytic reprogramming, lung fibrosis, matrix stiffness, PKM2

## Abstract

**Introduction:**

Matrix stiffening during idiopathic pulmonary fibrosis (IPF) creates a mechanically altered microenvironment that promotes fibroblast activation, yet the metabolic consequences of these mechanical cues remain incompletely defined. Pyruvate kinase M2 (PKM2) catalyzes the rate-limiting step of glycolysis, but whether it serves as a mechanosensitive link coupling matrix stiffness to fibroblast activation has not been clarified. This study examined whether substrate stiffness drives glycolytic reprogramming through PKM2 upregulation and whether this metabolic adaptation is required for mechanically driven fibroblast activation.

**Methods:**

PKM2 expression was analyzed in the NCBI GEO dataset GSE24206, human IPF lung tissues, and the bleomycin-induced mouse pulmonary fibrosis model. Primary mouse lung fibroblasts were cultured on collagen I–coated polyacrylamide hydrogels mimicking normal (2 kPa) or fibrotic (25 kPa) stiffness with or without TGF-β1 treatment. Glucose uptake, lactate secretion, pyruvate kinase activity, LDH activity, NAD^+^/NADH ratio, and oxygen consumption were measured; PKM2 oligomeric states were resolved by DSS cross-linking; and PKM2 was depleted by lentiviral shRNA.

**Results:**

PKM2 transcripts were progressively elevated in early- and advanced-stage IPF, and corresponding increases in protein and mRNA were confirmed in human IPF lungs and in bleomycin-treated mouse lungs. Stiff substrates upregulated PKM2 expression and reprogrammed fibroblast metabolism, with TGF-β1 eliciting increases in glucose uptake, lactate secretion, and oxygen consumption that were predominantly observed on stiff substrates. Cross-linking analysis showed that soft substrates favored catalytically active PKM2 tetramers, whereas stiff substrates constrained tetramer assembly and promoted lower-order species, providing a mechanistic basis for the dissociation between increased PKM2 expression and unchanged pyruvate kinase activity. PKM2 knockdown attenuated stiffness-induced increases in glucose uptake, lactate secretion, and LDH activity, reduced the cellular NAD^+^/NADH ratio, and decreased α-SMA–positive stress fiber formation under combined mechanical and TGF-β1 stimulation.

**Discussion:**

These findings indicate that PKM2 contributes to a mechanosensitive metabolic program coupling matrix stiffness to fibroblast activation, with substrate stiffness regulating both PKM2 expression and oligomeric state. Targeting PKM2-mediated metabolic adaptation may interrupt the self-reinforcing cycle of matrix stiffening and fibroblast activation in pulmonary fibrosis.

## Introduction

Idiopathic pulmonary fibrosis (IPF) is a progressive interstitial lung disease characterized by aberrant wound healing responses that result in excessive extracellular matrix deposition and ultimately respiratory failure (Kaminski et al., 2003; American Thoracic Society and European Respiratory Society, 2002; Katzenstein and Myers, 1998; Selman et al., 2001; Sheppard, 2001; McDonald, 1991). The disease affects approximately three million people worldwide, with median survival of 3–5 years from diagnosis (Hopkins et al., 2016; Koudstaal and Wijsenbeek, 2023; Glassberg, 2019). Therapeutic options are limited which moderately slow but do not halt disease progression (Raghu et al., 2022; Somogyi et al., 2019). A defining pathological feature of IPF is the progressive increase in lung tissue stiffness, where the elastic modulus rises from approximately 2 kPa in healthy lung parenchyma to over 20 kPa in fibrotic regions (Booth et al., 2012; Hinz, 2012; Freeberg et al., 2021). This mechanical alteration creates a fundamentally altered microenvironment that influences cellular behavior through mechanotransduction pathways, yet the metabolic consequences of these mechanical changes remain incompletely understood.

Tissue fibrosis develops through the transformation of resident fibroblasts into highly synthetic myofibroblasts (Hinz et al., 2007; Desmouliere et al., 2003). This activation process involves coordinated changes in cellular morphology, contractility, and biosynthetic capacity that require substantial metabolic adaptation to meet increased energetic and biosynthetic demands. While TGF-β1 signaling has been extensively characterized as a primary biochemical driver of fibroblast activation, emerging evidence indicates that mechanical cues from the stiffening matrix play equally important roles in maintaining and amplifying the fibrotic response (Wipff et al., 2007; Leight et al., 2012; Liu et al., 2010). The synergistic effect of biochemical and mechanical signals creates a self-reinforcing cycle where matrix stiffening promotes further fibroblast activation and matrix deposition, perpetuating disease progression even after initial injury resolution. Recent investigations have revealed that activated fibroblasts undergo metabolic reprogramming reminiscent of the Warburg effect observed in cancer cells, shifting from oxidative phosphorylation toward aerobic glycolysis despite adequate oxygen availability (Para et al., 2019; Xie et al., 2015). This metabolic adaptation provides rapid ATP generation, supplies biosynthetic precursors for matrix protein production, and maintains redox balance through NADPH generation required for cellular antioxidant systems. Pyruvate kinase M2 (PKM2) catalyzes the final rate-limiting step of glycolysis, converting phosphoenolpyruvate to pyruvate while generating ATP (Dayton et al., 2016; Israelsen and Vander Heiden, 2015; Shirai et al., 2016). Unlike the constitutively active PKM1 isoform, PKM2 exists in dynamic equilibrium between catalytically efficient tetramers and less active dimers that possess non-canonical functions including nuclear translocation and transcriptional regulation (Anastasiou et al., 2012; Wang et al., 2015). This unique regulatory flexibility positions PKM2 as a metabolic checkpoint that can rapidly adjust glycolytic flux in response to cellular demands while simultaneously modulating gene expression programs. PKM2 expression increases in various pathological conditions characterized by enhanced proliferation and biosynthesis, including cancer, inflammatory diseases, and tissue repair responses (Wong et al., 2013; Christofk et al., 2008). Previous studies have documented elevated PKM2 expression in IPF lung tissues and demonstrated anti-fibrotic effects of pharmacological PKM2 inhibition in bleomycin-induced pulmonary fibrosis models, establishing its involvement in fibrotic pathology (Gao et al., 2022; Han et al., 2021; Mei et al., 2022).

Despite recent advances, critical gaps remain in understanding how PKM2 contributes to fibroblast activation within the mechanically altered microenvironment of fibrotic tissue. Although PKM2 upregulation has been documented in fibrotic lungs, it is unclear whether this increase represents a downstream consequence of biochemical signaling, a direct response to altered matrix mechanics, or an integrated effect of both. The functional requirement of PKM2 in mechanically driven fibroblast activation has not been firmly established, leaving unresolved whether metabolic reprogramming actively enables or merely accompanies phenotypic transformation. Moreover, the possibility that PKM2 functions as a mechanosensitive metabolic regulator linking extracellular matrix stiffness to cellular behavior remains largely unexplored. These knowledge gaps hinder a full understanding of how biophysical cues shape fibrotic disease progression and limit the development of therapeutic strategies targeting mechanometabolic pathways. To address these questions, the present study tests the hypothesis that matrix stiffening drives glycolytic reprogramming through PKM2 upregulation and that this metabolic adaptation is essential for fibroblast activation. We investigated whether substrate rigidity regulates PKM2 expression and determined the functional consequences of disrupting this pathway. Using polyacrylamide hydrogels of defined stiffness to recapitulate the mechanical properties of normal and fibrotic lung tissue, we evaluated how matrix rigidity modulates fibroblast metabolism under controlled biochemical conditions. Through genetic loss-of-function approaches, we further examined whether PKM2 mediates the transduction of mechanical cues into fibrogenic cellular responses, thereby testing the broader concept that metabolic adaptation underlies mechanically driven pathological activation.

## Materials and methods

### Primary lung fibroblast culture and substrate engineering

Primary mouse lung fibroblasts were isolated as previously described and maintained in Dulbecco’s Modified Eagle Medium (DMEM) containing 5 mM glucose, 2 mM L-glutamine, 10% fetal bovine serum, and 1% penicillin/streptomycin at 37 °C with 5% CO_2_ (Edelman and Redente, 2018). Polyacrylamide hydrogel substrates were prepared using established acrylamide/bis-acrylamide formulations as previously described (Tse and Engler, 2010; Zhou et al., 2020; Pelham and Wang, 1997). Soft hydrogels (2 kPa) were prepared to mimic normal lung compliance, while stiff hydrogels (25 kPa) represent fibrotic tissue rigidity. All hydrogel surfaces were coated with rat-tail collagen I (0.1 mg/mL), ensuring consistent cell adhesion properties while preserving substrate mechanical characteristics (Tse and Engler, 2010; Zhou et al., 2020). Standard tissue culture polystyrene served as an ultra-stiff reference condition in selected experiments. Fibroblasts were seeded onto each substrate and allowed 24–48 h for complete attachment before experimental treatment. Myofibroblast differentiation was induced through treatment with recombinant mouse TGF-β1 (R&D Systems) for indicated hours. For long-term assessment, cells were maintained on hydrogels for 14 days with medium changes every 3 days before transfer to glass coverslips for immunofluorescence analysis, allowing visualization of stress fiber organization without hydrogel-associated autofluorescence.

### Lentiviral shRNA-mediated PKM2 silencing

Stable PKM2 knockdown was generated by lentiviral transduction using a validated short hairpin RNA construct (MISSION shRNA, Sigma-Aldrich; TRCN0000195605), with a scrambled shRNA serving as control. Lentiviral particles were produced as previously described (Brown et al., 2020), and transduced cells were selected with 1 μg/mL puromycin for 72 h to obtain stable knockdown populations. Knockdown efficiency was confirmed by Western blot analysis.

### Glucose uptake assay

Glucose uptake was measured using the 2-NBDG glucose uptake assay kit (Abcam, ab235976) following the manufacturer’s instructions and as described previously (Che et al., 2021). Briefly, cells were serum-starved in glucose-free medium for 2 h, then incubated with 100 µM 2-NBDG for 20 min at 37 °C. After incubation, cells were washed with ice-cold PBS to remove excess probe, and fluorescence intensity was measured using a plate reader (Ex/Em = 465/540 nm). Data was normalized to cell number and expressed as relative fold change to indicated control group.

### Lactate production assay

Lactate levels were measured using the L-lactate assay kit (Abcam, ab65331) according to the manufacturer’s instructions and as described previously (Che et al., 2021). Briefly, cells were incubated in phenol-red–free medium containing 5 mM glucose for 2 h, and the conditioned medium was centrifuged to remove debris. The supernatant was then analyzed colorimetrically at 570 nm using the supplied lactate oxidase reaction mix. Lactate concentrations were determined from standard curves and normalized to total protein content and expressed as relative fold change to indicated control group.

### Pyruvate kinase (PK) activity assay

Total pyruvate kinase activity was measured using pyruvate kinase assay kit (BioAssay Systems, #EPYR-100) according to the manufacturer’s instructions. Briefly, cell lysates were incubated with the provided reaction mix according to the manufacturer’s instructions. The decrease in absorbance at 340 nm was monitored using a microplate reader, and enzyme activity was calculated from the linear reaction rate and normalized to total protein content and expressed as relative fold change to indicated control group.

### Lactate dehydrogenase (LDH) activity assay

LDH activity was measured using the lactate dehydrogenase colorimetric activity kit (Invitrogen, #EEA013) following the manufacturer’s protocol. Briefly, cell lysates were incubated with the supplied reaction mix containing pyruvate and NADH, and the decrease in absorbance at 450 nm was monitored at 37 °C. Enzyme activity was calculated from the linear rate of NADH oxidation, normalized to total protein concentration, and expressed as relative fold change to indicated control group.

### Cellular redox state analysis

The intracellular NAD^+^/NADH ratio was measured using the NAD^+^/NADH assay kit (Abcam, ab65348) according to the manufacturer’s instructions. Briefly, cells were extracted using the provided NAD^+^ and NADH extraction buffers. After heat treatment to prevent interconversion, samples were neutralized and subjected to an enzymatic cycling reaction that converts NAD^+^/NADH to a colorimetric product. Absorbance was measured at 450 nm, and concentrations were calculated from standard curves. Final values were normalized to total protein content and expressed as relative fold change to indicated control group.

### Oxygen consumption rate assessment

Oxygen consumption rate was measured using oxygen consumption rate assay kit (Cayman Chemical, Item No. 600800) per the manufacturer’s instructions and as described previously (Che et al., 2021). Briefly, fibroblasts were incubated in assay buffer containing 10 mM glucose and 2 mM L-glutamine in 96-well plate. Following probe addition and plate sealing, fluorescence was recorded kinetically at 37 °C using excitation/emission settings of 380/650 nm. Data were analyzed using instrument-specific calibration to determine oxygen consumption rate, expressed as pmol O_2_ per minute per 10^4^ cells. Final values were normalized to total protein content determined and expressed as relative fold change to indicated control group.

### Quantitative gene expression analysis by RT-qPCR

Total RNA was extracted using the Monarch Total RNA Miniprep Kit (New England Biolabs) according to the manufacturer’s instructions and as described previously (Che et al., 2020). RNA purity and integrity were confirmed by A260/A280 absorbance ratio (>1.8). One microgram of total RNA was reverse-transcribed using the LunaScript® RT SuperMix Kit (New England Biolabs) with random hexamer primers. Quantitative PCR was performed using the Luna® Universal qPCR Master Mix (New England Biolabs) on CFX96 Real-Time PCR Detection System (Bio-Rad Laboratories). Primer efficiency and specificity were validated by melt-curve analysis. β-actin or GAPDH serves as internal reference genes. Relative mRNA expression levels were calculated using the 2^−ΔΔCt^ method and expressed as relative fold change to indicated control group.

### Protein expression analysis by Western blot

Whole cell lysates were prepared in radioimmunoprecipitation assay buffer supplemented with complete protease and phosphatase inhibitor cocktails to preserve protein integrity and phosphorylation states. Protein concentration was determined by BCA assay with bovine serum albumin standards. Equal total protein amounts (20 μg) underwent electrophoresis on 10% polyacrylamide gels, followed by transfer to polyvinylidene difluoride membranes as previously described (Che et al., 2021; Towbin et al., 1979). Membranes were blocked with 5% non-fat milk in Tris-buffered saline containing 0.1% Tween-20, then incubated overnight at 4 °C with indicated primary antibodies: PKM2 (Cell Signaling Technology #4053, 1:1000), α-SMA (Cell Signaling Technology #19245, 1:1000), and GAPDH (Cell Signaling Technology #2118, 1:1000), β-actin (Cell Signaling Technology #4967, 1:1000) as loading control. Following incubation with species-appropriate horseradish peroxidase-conjugated secondary antibodies, signals were detected using enhanced chemiluminescence.

### Cross-linking and detection of PKM2 oligomeric states

PKM2 oligomeric states were assessed by disuccinimidyl suberate (DSS) cross-linking followed by SDS-PAGE and Western blotting (Gao et al., 2022; Bahiraii et al., 2022; Yuan et al., 2020). Primary mouse lung fibroblasts were seeded on collagen I-coated polyacrylamide hydrogels (2 kPa or 25 kPa), allowed to attach overnight, serum-starved for 12 h, and treated with TGF-β1 (1, 2.5, or 5 ng/mL) or PBS for 60 h. At endpoint, cells were cross-linked with 2 mM DSS (Thermo Fisher, #21655) for 30 min at room temperature, quenched with 20 mM Tris-HCl (pH 7.5), and lysed in RIPA buffer (Bahiraii et al., 2022; Yuan et al., 2020). Equal total protein was resolved on 10% gels and immunoblotted as previously described. Equal loading was verified by GAPDH immunoblotting.

### Immunofluorescence microscopy

Cells cultured on hydrogel substrates or glass coverslips underwent fixation with 4% paraformaldehyde for 10 min, followed by permeabilization with 0.1% Triton X-100 and blocking with 5% bovine serum albumin to prevent non-specific binding. Primary antibodies against α-SMA (1:150 dilution) were applied overnight at 4 °C in humidified chambers. After extensive washing, samples were incubated with Alexa Fluor 488-conjugated secondary antibodies (1:1000) for 1 h at room temperature. Nuclei were counterstained using 4′,6-diamidino-2-phenylindole (DAPI). Images were acquired using a Nikon A1R Laser Scanning Confocal Microscope (Nikon Instruments Inc., Tokyo, Japan) with ×63 oil immersion objective, maintaining consistent laser power and gain settings across experimental groups. Quantification of α-SMA-positive cells exhibiting organized stress fibers was performed using ImageJ. At least 25 cells per condition were analyzed across three independent experiments, with image analysis conducted in a blinded manner.

### Bleomycin-induced pulmonary fibrosis model

Female C57BL/6 mice (The Jackson Laboratory, 8–12 weeks, 20–25 g) underwent single intratracheal instillation of bleomycin sulfate (3 U/kg body weight in 50 μL saline) or saline vehicle after being briefly anesthetized by isoflurane inhalation using the open-drop method following IACUC-approved protocols. At day 21 post-instillation, mice were euthanized by exsanguination under deep isoflurane anesthesia. The lungs were perfused with PBS via the right ventricle and then underwent inflation fixation at 25 cm H_2_O pressure with 4% paraformaldehyde for histological processing. Five-micrometer paraffin sections underwent staining with hematoxylin and eosin for general morphology, Masson’s trichrome for collagen visualization. Fibrosis severity was quantified using the modified Ashcroft scoring system by two independent blinded observers evaluating 20 random fields per animal. For lung homogenates, lung tissues were placed in pre-chilled tubes containing T-PER (Thermo Fisher Scientific) supplemented with Halt Protease Inhibitor Cocktail (Thermo Fisher Scientific) and homogenized on ice. The homogenates were then centrifuged at 9,000 × g for 10 min at 4 °C, and the resulting supernatants were collected into clean microcentrifuge tubes. Total protein concentrations were quantified using the BCA protein assay kit (Thermo Fisher Scientific).

### Statistical analysis

Statistical analyses were performed using GraphPad Prism 9.0. Differences among treatment groups were evaluated by one-way ANOVA followed by the Tukey’s *post hoc* test for multiple comparisons, or by two-tailed Student’s t-test when appropriate. Results are presented as mean ± standard error of the mean unless otherwise specified, with significance levels denoted as *p < 0.05, **p < 0.01, ***p < 0.001, while “ns” denotes no significant difference (*p* ≥ 0.05).

## Results

### PKM2 expression increases in human IPF lung tissue and activated lung fibroblasts

**FIGURE 1:**
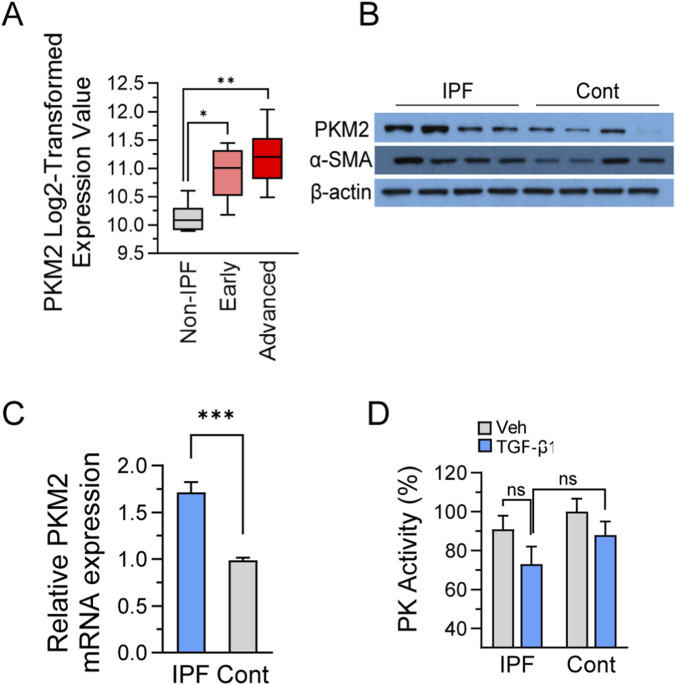
PKM2 expression is elevated in human IPF lung tissue and activated fibroblasts. **(A)** Bioinformatic analysis of PKM2 transcript levels from publicly available gene expression dataset comparing non-fibrotic control lung tissues (n = 6), early-stage IPF (n = 8), and advanced-stage IPF (n = 9). Data are presented as log2-transformed expression values. Box plots show median with interquartile range. **(B)** Western blot analysis of PKM2 protein expression in whole lung tissue homogenates from non-fibrotic control subjects (n = 5) and IPF patients (n = 5). Protein lysates (25 μg per lane) were separated by 10% SDS-PAGE and immunoblotted with anti-PKM2 antibody. β-actin served as loading control. **(C)** Quantitative real-time PCR analysis of PKM2 mRNA expression in total RNA extracted from control (n = 5) and IPF lung tissues (n = 5) with validated PKM2-specific primers. Gene expression was normalized to β-actin housekeeping gene using the 2^−ΔΔCt^ method. **(D)** Total pyruvate kinase enzymatic activity measured in cell lysates from primary lung fibroblasts treated with TGF-β1 (5 ng/mL) or PBS for 48 h using pyruvate kinase (PK) activity assay. Data represent mean ± SEM from three independent experiments. Statistical analyses were performed using an unpaired Student’s t-test or one-way ANOVA followed by Tukey’s *post hoc* test for multiple comparisons. *p < 0.05, **p < 0.01, ***p < 0.001. “ns” denotes no significant difference (*p* ≥ 0.05).

To establish the relevance of metabolic alterations in pulmonary fibrosis pathogenesis, we first examined PKM2 expression patterns across disease states. Analysis of the NCBI Gene Expression Omnibus dataset GSE24206, which contains gene expression profiles from healthy controls, early-stage, and advanced idiopathic pulmonary fibrosis lung tissues (Yang et al., 2013), revealed progressive upregulation of PKM2 transcripts correlating with disease severity. PKM2 expression increased significantly in early-stage IPF tissues compared to healthy controls, while advanced-stage samples showed a trend toward further elevation, though this incremental increase between early and advanced stages did not reach statistical significance (Figure 1A). This pattern suggests that metabolic reprogramming occurs relatively early in fibrosis development, with PKM2 upregulation potentially representing an initial adaptive response that plateaus as the disease progresses, rather than continuing to escalate throughout late-stage pathology. To determine whether these transcriptional changes translate to corresponding alterations in protein expression, we performed Western blot analysis on lung tissue homogenates obtained from IPF patients and non-fibrotic controls. PKM2 protein levels were consistently elevated in IPF samples, confirming that the observed transcriptional upregulation results in sustained protein accumulation (Figure 1B). Validation through quantitative PCR demonstrated a 1.7-fold elevation in PKM2 mRNA levels in IPF lung tissues compared to controls (Figure 1C). To assess whether increased PKM2 expression correlates with enhanced enzymatic function, we measured total pyruvate kinase activity in fibroblast lysates. It is important to note that PKM2 protein abundance and pyruvate kinase catalytic activity are not interchangeable measures: total PKM2 protein reflects all oligomeric forms, whereas catalytic activity is predominantly attributed to the tetrameric configuration. Interestingly, despite the robust upregulation of PKM2 protein and mRNA, TGF-β1 treatment did not result in increased pyruvate kinase activity in both IPF and control fibroblasts (Figure 1D). This apparent discordance between protein abundance and catalytic activity aligns with established mechanisms whereby PKM2 undergoes oligomeric transitions from highly active tetramers to less active dimers during cellular stress and metabolic reprogramming. Collectively, these results establish that PKM2 undergoes progressive upregulation in human pulmonary fibrosis, with expression levels correlating with disease severity. The dissociation between PKM2 protein abundance and enzymatic activity represents a regulated metabolic switch rather than a simple gain of catalytic function.

### PKM2 expression is elevated in bleomycin-induced experimental pulmonary fibrosis

**FIGURE 2:**
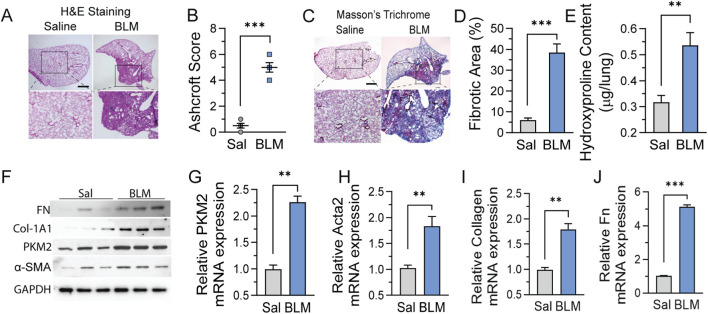
PKM2 expression is elevated in bleomycin-induced experimental pulmonary fibrosis. Lung histology from C57BL/6 mice 21 days following intratracheal administration of bleomycin (3 U/kg body weight) or equivalent volume saline vehicle control. **(A)** Mouse lung sections (5 μm) were stained with hematoxylin and eosin (H&E) to assess tissue architecture. Representative images are shown with boxed regions indicating areas enlarged below. Scale bar = 1000 μm. **(B)** Modified Ashcroft scoring for quantitative assessment of fibrosis severity performed by two blinded investigators evaluating 20 random non-overlapping fields per mouse at 200× magnification. Scoring scale ranges from 0 (normal lung) to 8 (complete obliteration of fields). **(C)** Representative Masson’s trichrome staining demonstrating collagen deposition (blue) from saline and bleomycin-treated mice. Representative images are shown with boxed regions indicating areas enlarged below. Scale bar = 1000 μm. **(D)** Morphometric quantification of fibrotic area percentage from trichrome-stained sections using ImageJ software with color threshold analysis. **(E)** Biochemical quantification of lung hydroxyproline content. **(F)** Western blot analysis of PKM2, α-SMA, Collagen I, and Fibronectin protein expression in whole lung homogenates from saline control and bleomycin-treated mice. GAPDH served as loading control. **(G–J)** Quantitative real-time PCR analysis of **(G)** PKM2, **(H)** Acta2 **(I)** Col1a1, and **(J)** Fn1 mRNA expression in total lung RNA. Gene expression normalized to β-actin using the 2^−ΔΔCt^ method and expressed as fold change relative to saline controls. Data represent mean ± SEM with n = 3 mice per group. Statistical significance determined by unpaired Student’s t-test. **p < 0.01, ***p < 0.001.

To determine whether PKM2 upregulation represents a conserved fibrotic response across species, we examined its expression in the well-established bleomycin-induced pulmonary fibrosis model. C57BL/6 mice received a single intratracheal instillation of bleomycin and were analyzed at day 21, corresponding to peak fibrotic response in this model system. Hematoxylin and eosin staining revealed marked architectural distortion in bleomycin-treated lungs, characterized by alveolar wall thickening and obliteration of normal airspace architecture, contrasting sharply with the preserved alveolar structure and thin septa observed in saline-treated controls (Figure 2A). Blinded quantification using the modified Ashcroft scoring system confirmed severe fibrosis, with bleomycin-treated animals achieving mean scores of 4.8 compared to 0.5 in controls, indicating extensive parenchymal remodeling (Figure 2B). Masson’s trichrome staining demonstrated collagen accumulation throughout fibrotic lung regions, appearing as extensive blue-stained regions that replaced normal alveolar architecture, whereas control lungs exhibited minimal collagen restricted to perivascular regions (Figure 2C). Morphometric analysis using standardized color-threshold segmentation revealed that fibrotic areas occupied 38.3% of total lung area in bleomycin-treated mice versus 6.2% in controls, confirming elevated matrix deposition (Figure 2D). These histological findings were corroborated by biochemical measurement of hydroxyproline content, a specific marker of collagen abundance. Total lung hydroxyproline increased from 0.31 μg/lung in saline controls to 0.52 μg/lung in bleomycin-treated mice (Figure 2E). We next assessed PKM2 expression to examine whether metabolic reprogramming accompanies matrix remodeling in experimental fibrosis. Western blot analysis of whole-lung homogenates revealed substantial PKM2 protein upregulation in bleomycin-treated mice, accompanied by increased expression of α-smooth muscle actin, Collagen I, and Fibronectin (Figure 2F). The coordinate elevation of these myofibroblast differentiation markers and extracellular matrix proteins provides direct immunoblot evidence for both fibroblast activation and pathological matrix accumulation in this model. Quantitative PCR analysis validated these protein level changes, showing PKM2 mRNA levels increased in fibrotic lungs (Figure 2G), accompanied by elevation of the myofibroblast differentiation marker Acta2 (Figure 2H), the extracellular matrix gene Col1a1 and Fn1 (Figures 2I,J). The concordance between protein and mRNA data across multiple fibrotic markers together with the histological and biochemical collagen assessments (Figures 2A–E), provides a comprehensive multi-modal characterization of both myofibroblast activation and extracellular matrix remodeling in the bleomycin-induced fibrosis model.

### Stiff substrates upregulate PKM2 expression and metabolic reprogramming in primary mouse lung fibroblasts

Having established that PKM2 expression increases in fibrotic lung tissue, we next investigated whether the mechanical properties of the extracellular matrix directly influence PKM2 regulation and cellular metabolism. This question holds relevance given that tissue stiffening represents a defining characteristic of pulmonary fibrosis, with fibrotic regions exhibiting mechanical properties markedly distinct from healthy lung parenchyma. To model the mechanical microenvironment encountered by fibroblasts during fibrosis progression, we employed collagen-coated polyacrylamide hydrogels engineered to recapitulate pathologically relevant tissue stiffnesses: 2 kPa approximating healthy lung tissue and 25 kPa representing the increased rigidity characteristic of fibrotic regions.

**FIGURE 3:**
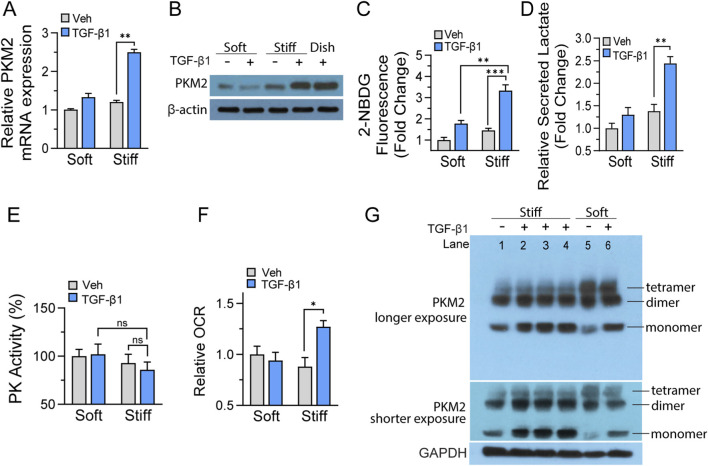
Stiff substrates upregulate PKM2 expression and metabolic reprogramming in primary mouse lung fibroblasts. **(A,B)** Primary mouse lung fibroblasts cultured on collagen I-coated polyacrylamide hydrogels of defined stiffness (2 kPa mimicking normal lung; 25 kPa mimicking fibrotic lung) or tissue culture plastic for 60 h in the presence of TGF-β1 (5 ng/mL). **(A)** Quantitative real-time PCR analysis of PKM2 mRNA expression in fibroblasts cultured on substrates of indicated stiffness. RNA was extracted using Monarch Total RNA Miniprep Kit, reverse transcribed, and amplified using validated PKM2 primers. Gene expression normalized to β-actin using the 2^−ΔΔCT^ method and expressed as fold change relative to the soft substrate (2 kPa) + PBS vehicle control. **(B)** Western blot analysis of PKM2 protein expression in whole cell lysates prepared in RIPA buffer containing protease and phosphatase inhibitors. **(C)** Glucose uptake was assessed using the fluorescent glucose analog 2-NBDG in fibroblasts cultured on indicated stiffness. Cells were transferred into 96-well plates and were allowed adhesion before reading. Fluorescence intensity was measured and normalized to cell number and expressed as relative fold change to indicated control group. **(D)** Lactate production quantified from culture supernatants using lactate assay under same culture conditions. **(E)** Pyruvate kinase enzymatic activity was measured in fibroblast lysates using a colorimetric assay based on NADH oxidation. Cell lysates were prepared after 60 h of TGF-β1 stimulation, and enzyme activity was quantified by monitoring the decrease in absorbance at 340 nm, normalized to total protein concentration. **(F)** Oxygen consumption rate (OCR) was measured using an OCR assay kit in fibroblasts cultured on collagen-fibroblasts cultured on collagen I-coated hydrogels of indicated stiffness. Cells were incubated in assay buffer, followed by addition of the oxygen-sensitive probe and plate sealing. Fluorescence was recorded kinetically at 37 °C (Ex/Em = 380/650 nm), and OCR was calculated from calibration standards and expressed as relative fold change to indicated control group. Data represents SEM with n = 3-4 per group. **(G)** Cross-linking analysis of PKM2 oligomeric states in primary mouse lung fibroblasts. Cells were cultured on collagen I-coated polyacrylamide hydrogels of defined stiffness (25 kPa or 2 kPa) for 60 h in the presence of TGF-β1 at indicated concentrations or PBS vehicle control. Cells were cross-linked with DSS, lysed, and protein lysates were separated by SDS-PAGE and immunoblotted with anti-PKM2 antibody. Both long-exposure and short-exposure films are shown to help visualization of tetrameric and dimeric bands, respectively. Bands corresponding to PKM2 monomer (∼60 kDa), dimer (∼120 kDa), and tetramer (∼240 kDa) are indicated. Lane 1: 25 kPa + PBS; lane 2: 25 kPa + TGF-β1 (1 ng/mL); lane 3: 25 kPa + TGF-β1 (2.5 ng/mL); lane 4: 25 kPa + TGF-β1 (5 ng/mL); lane 5: 2 kPa + PBS; lane 6: 2 kPa + TGF-β1 (5 ng/mL). Equal protein loading was verified by BCA assay and GAPDH immunoblotting. Statistical significance determined by unpaired Student’s t-test. *p < 0.05, **p < 0.01, ***p < 0.001. “ns” denotes no significant difference (*p* ≥ 0.05).

Primary mouse lung fibroblasts were cultured on these mechanically defined substrates for 60 h in the presence of TGF-β1, thereby combining the mechanical and biochemical stimuli which synergistically drive fibroblast activation *in vivo*. Quantitative RT-PCR analysis revealed that substrate stiffness profoundly influenced PKM2 transcriptional regulation, with expression levels increasing in a stiffness-dependent manner (Figure 3A). Fibroblasts cultured on stiff hydrogels exhibited significantly elevated PKM2 mRNA compared to those on soft substrates, while cells on tissue culture plastic showed comparable upregulation, suggesting that PKM2 transcriptional response plateaus at supraphysiological stiffness levels. These transcriptional changes were aligned with protein expression, as Western blot analysis demonstrated robust PKM2 protein accumulation in fibroblasts exposed to increased substrate rigidity (Figure 3B). Conventional tissue culture plastic served as a reference condition.

To determine whether stiffness-induced PKM2 upregulation produces functional metabolic consequences, we examined metabolic profiles of fibroblasts cultured on soft versus stiff substrates with or without TGF-β1 stimulation. Glucose consumption measurements using 2-NBDG uptake assays revealed increases in glucose utilization that correlated positively with substrate stiffness. Fibroblasts on soft hydrogels maintained relatively modest glucose consumption rates, while those on stiff substrates demonstrated significantly enhanced uptake in response to TGF-β1 (Figure 3C). This enhanced glucose uptake was metabolically coupled with increased lactate production (Figure 3D). Notably, total pyruvate kinase enzymatic activity measurements revealed a not statistically significant but declining trend in catalytic function on stiff substrate (Figure 3E). This unchanged catalytic activity likely reflects conformational transitions favoring less active PKM2 dimers over highly active tetramers. To assess the broader metabolic consequences of stiffness-induced changes, we measured cellular oxygen consumption rates. Basal mitochondrial respiration remained comparable between fibroblasts cultured on soft and stiff substrates under unstimulated conditions, indicating that substrate rigidity alone does not alter oxidative metabolism in the absence of additional profibrotic signals. However, TGF-β1 stimulation revealed a substrate-dependent metabolic divergence that underscores the synergistic effect between mechanical and biochemical cues. Specifically, fibroblasts cultured on stiff substrates demonstrated a 34% increase in mitochondrial respiration following TGF-β1 treatment, compared to PBS-treated control. In contrast, fibroblasts cultured on soft matrices showed no appreciable change in respiratory capacity despite exposure to the same concentration and duration of TGF-β1 stimulation (Figure 3F). This substrate stiffness-dependent enhancement of oxidative phosphorylation reveals that mechanical cues serve as critical modulators of cellular metabolic responsiveness to biochemical signals.

To determine whether the mechanical microenvironment influences PKM2 oligomeric configuration, we performed chemical cross-linking analysis resolving monomeric (∼60 kDa), dimeric (∼120 kDa), and tetrameric (∼240 kDa) PKM2 forms (Figure 3G). Primary lung fibroblasts were cultured on stiff (25 kPa) or soft (2 kPa) collagen I coated polyacrylamide hydrogels, treated with TGF-β1 or PBS for 60 h, and analyzed by Western blot. On stiff substrates, the tetrameric pool was uniformly low across all four conditions (lanes 1–4) regardless of TGF-β1 dose. TGF-β1 treatment increased monomeric PKM2 (lanes 2–4 versus lane 1), and shorter-exposure films revealed a modest but consistent increase in dimeric PKM2 in these same lanes, indicating that the additional PKM2 induced by TGF-β1 on stiff substrates accumulated as lower-order species rather than forming tetramers. Soft substrates displayed a markedly different pattern. The tetrameric pool was substantially elevated in both PBS (lane 5) and TGF-β1 treated (lane 6) conditions, while lane 5 exhibited the lowest monomer of all six conditions. The dimeric fraction on soft substrates (lanes 5–6) remained lower than TGF-β1-treated stiff conditions (lanes 2–4), indicating that dimer accumulation was specific response to stiff substrates. Together, these data demonstrate that substrate stiffness is a dominant determinant of PKM2 oligomeric distribution, with stiff substrates constraining tetramer formation and soft substrates favoring it. This stiffness-dependent difference in oligomeric assembly directly accounts for the dissociation between PKM2 expression and enzymatic activity (Figures 3B,E), as increased PKM2 protein on stiff substrates did not translate into proportionally increased catalytically active tetramers.

Collectively, these findings establish that matrix stiffness functions as a critical regulator of fibroblast metabolism through mechanisms involving PKM2 upregulation and functional modulation. The observation that increased substrate rigidity drives PKM2 expression while pyruvate kinase activity did not increase proportionally highlights the complexity of metabolic adaptation to mechanical cues. These results position PKM2 as a mechanosensitive metabolic switch that translates physical properties of the extracellular matrix into cellular metabolic programs.

### PKM2 contributes to myofibroblast activation on stiff substrates

**FIGURE 4:**
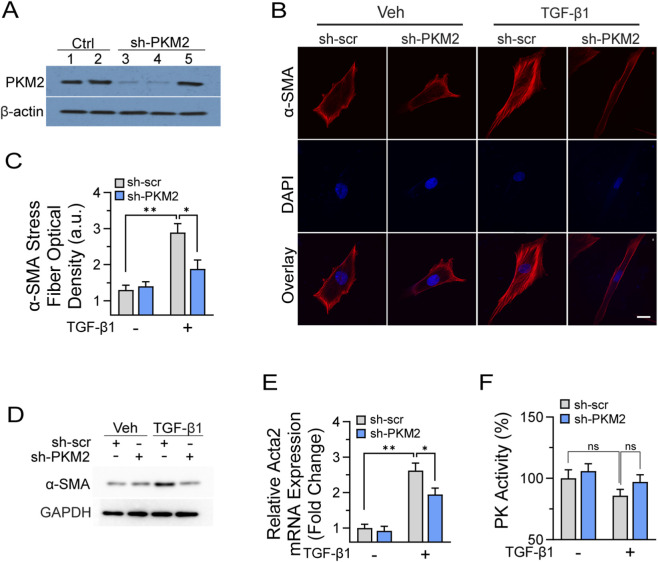
PKM2 contributes to myofibroblast activation on stiff substrate. **(A)** Validation of lentiviral shRNA-mediated PKM2 knockdown efficiency. Primary mouse lung fibroblasts were transduced with lentivirus encoding PKM2-targeted shRNA or scrambled control shRNA, followed by puromycin selection. Western blot analysis showed PKM2 protein levels with β-actin as loading control. (Lane 1, mock infected; lane 2, sh-scr; lane 3, sh-PKM2-1; lane 4, sh-PKM2-2; lane 5, sh-PKM2-3). **(B)** Representative immunofluorescence microscopy images showing α-smooth muscle actin (red fluorescence) in sh-scr control and PKM2-knockdown fibroblasts. Fibroblasts were cultured on 25 kPa polyacrylamide hydrogels for 14 days with TGF-β1 (5 ng/mL), then transferred to glass coverslips, fixed in 4% paraformaldehyde, and immunostained with anti-α-SMA antibody followed by Alexa Fluor 488-conjugated secondary antibody. Nuclei was counterstained with DAPI. Scale bars = 20 μm. **(C)** Quantification of α-SMA stress fiber optical density per cell from immunofluorescence images using ImageJ. At least 25 cells per condition were analyzed across three independent experiments in a blinded manner. Data are expressed in arbitrary units (a.u.). **(D)** Western blot analysis of α-SMA protein expression in scramble control and PKM2-knockdown fibroblasts cultured on 25 kPa substrates for 60 h with TGF-β1 treatment. **(E)** Quantitative real-time PCR analysis of Acta2 mRNA expression under identical culture conditions, normalized to β-actin and expressed as relative fold change. **(F)** Total pyruvate kinase enzymatic activity measured in cell lysates from scrambled control and PKM2 knockdown fibroblasts using coupled enzyme assay. Data represent mean ± SEM from 3 to 4 independent experiments. Statistical analyses were performed using an unpaired Student’s t-test or one-way ANOVA followed by Tukey’s *post hoc* test for multiple comparisons. *p < 0.05, **p < 0.01, ***p < 0.001. “ns” denotes no significant difference (p ≥ 0.05).

Myofibroblast differentiation represents a defining hallmark of fibrogenesis, with mechanical stiffness serving as a potent driver of this phenotypic transition through well-characterized mechanotransduction pathways (Hinz, 2012; Wipff et al., 2007; Tomasek et al., 2002). Having established that PKM2 expression increases in response to both biochemical and mechanical cues, we next investigated whether PKM2 functionally contributes to stiffness-induced myofibroblast differentiation. To address this question, we employed a loss-of-function approach using lentiviral-mediated shRNA delivery to achieve stable PKM2 knockdown in primary lung fibroblasts. This genetic manipulation resulted in robust suppression of PKM2 expression, with protein levels (sh-PKM2-1, lane 3; sh-PKM2-2, lane 4) were reduced by approximately 90% compared to mock-infected (lane 1) and scrambled control shRNA (sh-scr, lane 2) as verified by Western blot analysis (Figure 4A). The sustained knockdown efficiency over the extended culture period was confirmed at multiple timepoints, ensuring consistent PKM2 depletion throughout the differentiation assays.

To evaluate the functional consequences of PKM2 loss on mechanically driven fibroblast activation, we cultured transduced cells on collagen-coated polyacrylamide hydrogels calibrated to 25 kPa stiffness. Cells were maintained under TGF-β1 stimulation for 14 days to recapitulate the chronic biochemical and mechanical signals characteristic of the fibrotic microenvironment. This extended culture period allows for complete phenotypic transition and stable myofibroblast differentiation. Immunofluorescence analysis revealed that sh-scr control fibroblasts underwent extensive myofibroblast differentiation in response to the combined mechanical and biochemical stimuli, with prominent α-SMA incorporation into organized stress fibers spanning the cell body. Quantitative image analysis demonstrated that TGF-β1-treated sh-scr fibroblasts exhibited over 2-fold higher α-SMA stress fiber density compared with vehicle-treated sh-scr cells, whereas this induction was markedly reduced in PKM2-knockdown fibroblasts (Figures 4B,C). Importantly, while PKM2 knockdown modestly reduced proliferation rates in fibroblasts cultured on stiff substrates, it did not compromise cell viability or substrate adhesion capacity. Western blot analysis corroborated the immunofluorescence findings, demonstrating that PKM2 knockdown significantly attenuated α-SMA protein accumulation in response to TGF-β1 treatment on stiff substrates (Figure 4D). This substantial but incomplete suppression suggests that while PKM2 plays an important role in myofibroblast differentiation, parallel pathways independent of PKM2-mediated metabolism can partially compensate for its loss. Quantitative PCR analysis demonstrated corresponding reductions in ACTA2 mRNA levels (Figure 4E). To investigate whether the effects of PKM2 knockdown on myofibroblast differentiation stemmed primarily from loss of pyruvate kinase enzymatic activity, we measured total cellular pyruvate kinase activity in lysates from control and PKM2-deficient fibroblasts. Basal level pyruvate kinase activity declined following TGF-β1 stimulation, consistent with the reduced activity observed in IPF fibroblasts (Figure 1D). Notably, PKM2 depletion moderately restored pyruvate kinase activity under the same conditions (Figure 4F). Collectively, these findings establish PKM2 as an important contributory factor in mechanically induced myofibroblast differentiation. The substantial attenuation of α-SMA expression and stress fiber formation following PKM2 knockdown identifies this metabolic regulator as a significant node linking mechanical signals to cellular phenotypic transitions.

### PKM2 is required for the glycolytic shift driven by matrix stiffness and TGF-β1

**FIGURE 5:**
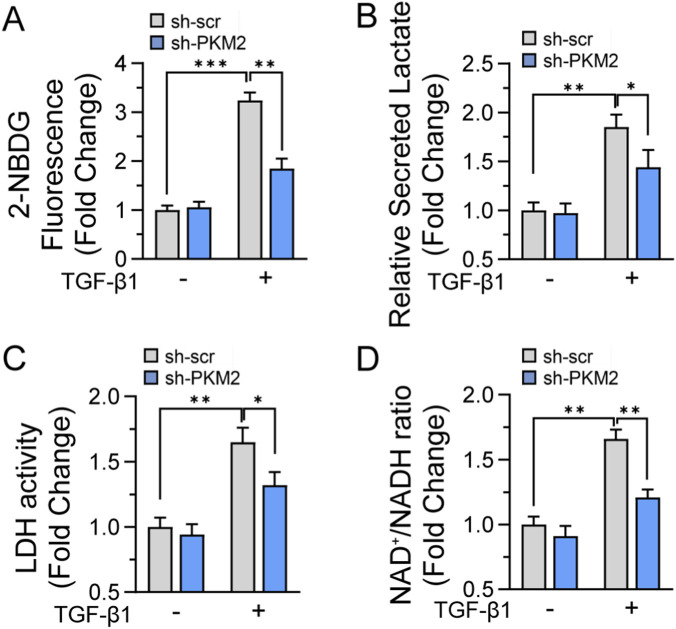
PKM2 is required for the glycolytic shift driven by matrix stiffness and TGF-β1. Metabolic flux analyses were performed in scrambled control and PKM2-knockdown fibroblasts cultured on collagen-coated 25 kPa hydrogels for 60 h in the presence of TGF-β1 (5 ng/mL) to model fibrotic mechanical and biochemical conditions. **(A)** Glucose uptake was assessed using the fluorescent glucose analog 2-NBDG in fibroblasts cultured on indicated stiffness. Cells were transferred into 96-well plates and were allowed adhesion before reading. Fluorescence intensity was measured and normalized to cell number. **(B)** Lactate production is quantified from culture supernatants using lactate assay under same culture conditions. **(C)** Lactate dehydrogenase (LDH) enzymatic activity measured in cell lysates using pyruvate-dependent NADH oxidation assay monitored spectrophotometrically at 340 nm. Activity normalized to total protein content and expressed fold changed. **(D)** Cellular NADH/NAD+ ratio determined by enzymatic cycling assay. Ratio calculated from independent measurements of NADH and total NAD pools, with values normalized to protein content. Data represent mean ± SEM from 4 to 5 independent experiments. Statistical analyses were performed using an unpaired Student’s t-test or one-way ANOVA followed by Tukey’s *post hoc* test for multiple comparisons. *p < 0.05, **p < 0.01, ***p < 0.001.

To determine whether PKM2 deficiency disrupts the metabolic adaptations characteristic of fibroblasts responding to mechanical cues, we examined metabolic profiles of PKM2-knockdown cells cultured on pathologically stiff substrates (25 kPa) in the presence of TGF-β1. Glucose uptake measurements revealed that PKM2 knockdown significantly attenuated TGF-β1 induced glucose consumption compared to scrambled control cells (Figure 5A). This marked reduction in glucose utilization was accompanied by a corresponding decrease in lactate secretion, with PKM2-deficient fibroblasts producing significantly less lactate than control cells in response to TGF-β1 stimulation (Figure 5B). To elucidate the mechanistic basis for the observed metabolic disruption in PKM2-deficient cells, we examined lactate dehydrogenase activity, the terminal glycolytic enzyme that catalyzes the reversible conversion of pyruvate to lactate while regenerating the NAD+ cofactor essential for sustained glycolytic flux. As anticipated for metabolically activated fibroblasts, TGF-β1 treatment of control cells cultured on stiff substrates (25 kPa) induced a significant increase in LDH activity compared to PBS-treated controls, demonstrating approximately 1.6 fold elevation, consistent with the enhanced glycolytic capacity characteristic of the profibrotic phenotype. In contrast, PKM2-depleted fibroblasts exhibited significantly attenuated LDH activity under identical conditions, showing approximately 23% lower enzyme activity compared to scrambled control cells receiving TGF-β1 treatment on stiff matrices (Figure 5C). This reduction in LDH activity, while not completely ablating enzyme function, represents a metabolic constraint that likely contributes to the impaired glycolytic capacity observed in PKM2-deficient cells. Measurement of the NAD^+^/NADH ratio, a key indicator of cellular redox state and metabolic flux, revealed that PKM2-knockdown cells exhibited over 25% reduction compared to control cells when cultured on stiff substrates with TGF-β1 treatment (Figure 5D). Under normal fibroblast activation conditions, cells maintain redox homeostasis through the coordinated regulation of multiple NAD^+^ regeneration pathways, including the lactate dehydrogenase reaction in the cytosol and the electron transport chain in mitochondria. The inability of PKM2-deficient cells to maintain appropriate NAD^+^/NADH balance despite potential compensatory mechanisms suggests that PKM2 plays a fundamental role in coordinating cellular redox state with the metabolic demands of the activated phenotype. Collectively, these findings reveal that PKM2 deficiency creates a cascade of metabolic perturbations that compromise the stiff substrate driven metabolic rewiring that is necessary for both energy generation and biosynthetic processes under profibrotic conditions.

## Discussion

This study demonstrates that stiff matrix promotes metabolic reprogramming through PKM2 upregulation, establishing a functional connection between the mechanical properties of fibrotic tissue and lung fibroblast activation. Our analysis of PKM2 expressions in human IPF tissues, mouse lung fibrotic lungs, and hydrogel culture systems reveal that PKM2 plays an important role in stiffness-induced fibroblast activation. The progressive increase in PKM2 expression observed in human IPF tissues aligns with previous analyses of the GSE24206 dataset. While our analysis detected significant upregulation in both early and advanced disease stages, the precise expression changes require further validation across larger patient cohorts. The parallel elevation of PKM2 protein in bleomycin-treated mouse lungs confirms that this metabolic alteration represents a conserved fibrotic response across species. A particularly intriguing finding involves the dissociation between PKM2 protein abundance and enzymatic activity. Despite increased mRNA and protein expression on stiff substrates and following TGF-β1 treatment, pyruvate kinase activity did not increase proportionally. Our cross-linking analysis provides biochemical evidence for this relationship and reveals a key role for the mechanical microenvironment. On soft substrates, the tetrameric pool was robustly elevated under both unstimulated and TGF-β1 treated conditions, with the monomeric pool at its lowest under unstimulated conditions and only modestly increased by TGF-β1 treatment, and the dimeric fraction at baseline. To our knowledge, this represents the first biochemical resolution of PKM2 oligomeric states on physiologically compliant substrates, as all prior cross-linking analyses in fibroblasts were conducted on rigid tissue culture plastic. Gao et al. reported elevated PKM2 tetramer in fibroblasts isolated from bleomycin-induced fibrotic lungs (Gao et al., 2022); however, those cells were replated on plastic for analysis, precluding assessment of how substrate compliance regulates oligomeric assembly *in situ*. Our data demonstrate that a compliant mechanical environment intrinsically favors tetramer assembly, potentially corresponding to the early stages of fibrotic tissue remodeling before substantial matrix stiffening has occurred. On stiff substrates, the additional PKM2 protein accumulated predominantly as monomers and dimers, while the tetrameric pool remained uniformly low across the TGF-β1 dose-response (1–5 ng/mL). The TGF-β1 induced dimer increase on stiff substrates is consistent with reports on rigid plastic, where TGF-β treatment promoted PKM2 dimerization and where stiff matrix was reported to facilitate PKM2 dimerization (Satyanarayana et al., 2021; Zhang et al., 2023). However, the magnitude of the dimer shift in our system was more modest, which likely reflects the substantially lower stiffness of our 25 kPa hydrogels compared to tissue culture plastic. Additional experimental variables including hydrogel surface chemistry, cross-linking reagent specificity (DSS versus glutaraldehyde), and cell type may further contribute to differences in absolute dimer magnitude. Importantly, TGF-β1 did not increase dimer levels on soft substrates (lanes 5 and 6), in contrast to the dimer accumulation observed on stiff substrates. This observation indicates that a normal physiological mechanical environment maintains conditions that favor tetramer assembly. Regardless of the lower-order species that accumulates on stiff substrates, the functional consequence is the same: reduced tetramer formation results in decreased per-unit pyruvate kinase activity. This apparent paradox that increased PKM2 expression without proportionally increased kinase activity therefore reflects the dual nature of PKM2 function: reduced enzymatic activity diverts glycolytic intermediates toward biosynthetic pathways supporting collagen production, while monomeric and dimeric PKM2 may gain non-metabolic functions including nuclear translocation and transcriptional regulation (Yang et al., 2011; Luo et al., 2011; Selvarajah et al., 2021; Warburg, 1956; Potter et al., 2016; Liberti and Locasale, 2016).

The therapeutic implications of our findings warrant careful consideration in light of the emerging complexity of PKM2 biology in organ fibrosis. While our data demonstrate that PKM2 depletion attenuates mechanically driven glycolytic reprogramming and myofibroblast differentiation, pharmacological targeting of PKM2 in pulmonary fibrosis is complicated by the context-dependent functions of its oligomeric forms. Gao et al. demonstrated that the PKM2 tetramer activator TEPP-46 paradoxically exacerbated bleomycin-induced pulmonary fibrosis through a non-metabolic mechanism: PKM2 tetramers directly bind Smad7 at its MH2 domain, sequestering Smad7 from TβRI and thereby amplifying TGF-β1 signaling (Gao et al., 2022). Conversely, TEPP-46 has shown protective effects in kidney, liver, and cardiac fibrosis models (Qi et al., 2017; Liu et al., 2021; Zheng et al., 2020). This organ-specific dichotomy underscores that PKM2 oligomeric forms may serve dual functions whose relative contributions vary across tissue microenvironments. Our oligomeric data add further nuance by revealing that the capacity for TGF-β1-driven tetramer assembly is itself affected by substrate stiffness, suggesting that the balance between metabolic and non-metabolic PKM2 functions may shift as the fibrotic microenvironment evolves. Approaches that selectively disrupt the metabolic output of dimeric PKM2 without altering the tetrameric pool may offer a more precise therapeutic window.

Several limitations temper our interpretations. Polyacrylamide hydrogels, while providing precise mechanical control, cannot recapitulate the complex three-dimensional architecture, heterogeneous cell populations, and dynamic viscoelastic properties of native lung tissue. The elastic behavior of synthetic substrates differs fundamentally from the time-dependent mechanical properties of fibrotic tissue, where stress relaxation and matrix remodeling continuously alter the mechanical environment. Additionally, our focus on fibroblasts, though mechanistically informative, overlooks potential metabolic changes in epithelial, endothelial, and immune cells that collectively drive fibrosis progression. Future studies incorporating organ-on-chip models or precision-cut lung slices may better capture these multicellular interactions. Furthermore, while our loss-of-function approach establishes the necessity of PKM2 for mechanically driven glycolytic reprogramming, the current study lacks complementary pharmacological validation and genetic gain-of-function data. Available PKM2 tetramer activators such as TEPP-46 are unsuitable for the lung due to confounding pro-fibrotic effects via Smad7-dependent TβRI stabilization (Gao et al., 2022). Future studies employing PKM2 inhibitors (e.g., shikonin) and catalytically dead mutants (e.g., K270M) will be essential for fully dissecting metabolic versus non-metabolic contributions. Future systematic analysis across a broader stiffness range would also help define the mechanical thresholds governing PKM2 oligomeric regulation.

Several important experimental directions emerge from these findings, each of which extends beyond the scope of the present study but represents an immediate priority for follow-up investigation. First, shikonin, which inhibits PKM2 enzymatic activity without the tetramer-dependent Smad7 confound, represents a candidate for pharmacological validation (Zhang et al., 2017). Second, catalytically dead PKM2 mutants could dissect metabolic versus non-metabolic functions: if catalytically dead PKM2 mutants fails to rescue glycolysis in PKM2-depleted cells while promoting myofibroblast differentiation through TβRI stabilization, this would establish metabolic output as the critical mediator. Third, upstream mechanotransduction pathways connecting matrix stiffness to PKM2 oligomeric redistribution require elucidation. Finally, systematic stiffness-range analysis would clarify whether the impairment of tetramer assembly reflects a threshold effect or a graded response during fibrosis progression.

In conclusion, our findings establish PKM2-mediated glycolytic reprogramming as a significant contributory mechanism in mechanically induced fibroblast activation. Cross-linking analysis reveals that the mechanical microenvironment critically regulates the oligomeric fate of newly expressed PKM2: soft substrates robustly support tetramer assembly and preserve catalytic activity, whereas stiff substrates constrain tetramer formation, causing the additional PKM2 to accumulate predominantly as catalytically inactive monomers with a modest TGF-β1 dependent dimer increase. This stiffness-dependent constraint on tetramer assembly provides a direct mechanistic explanation for the paradoxical dissociation between increased PKM2 expression and declining per-unit kinase activity, explaining how substrate stiffness reprograms glycolytic flux independent of total protein abundance. Our observation that PKM2 oligomeric regulation on physiologically compliant substrates differs from published observations on rigid plastic highlights the importance of mechanically defined culture systems for studying metabolic enzymes whose function depends on oligomeric state. This has broader implications given that PKM2 tetramers serve distinct non-metabolic functions, including Smad7 sequestration and TβRI stabilization in the lung. The partial but meaningful reduction in fibrotic responses following PKM2 knockdown positions this metabolic node as a potential therapeutic target that could complement existing antifibrotic strategies. Future investigations should focus on defining precise mechanical thresholds, dissecting metabolic from non-metabolic PKM2 functions through catalytic mutants and selective inhibitors, and evaluating combination therapies targeting multiple nodes within the mechanical–metabolic signaling network.

## Data Availability

The original contributions presented in the study are included in the article/supplementary material, further inquiries can be directed to the corresponding author.
